# Association between Fcγ receptor IIA, IIIA and IIIB genetic polymorphisms and susceptibility to severe malaria anemia in children in western Kenya

**DOI:** 10.1186/s12879-017-2390-0

**Published:** 2017-04-20

**Authors:** Elly O. Munde, Winnie A. Okeyo, Evans Raballah, Samuel B. Anyona, Tom Were, John M. Ong’echa, Douglas J. Perkins, Collins Ouma

**Affiliations:** 1 Department of Biomedical Sciences and Technology, School of Public Health and Community Development, Maseno University, Maseno, Kenya; 2 University of New Mexico/KEMRI Laboratories of Parasitic and Viral Diseases, Centre for Global Health Research, Kenya Medical Research Institute, Kisumu, Kenya; 3 Department of Medical Laboratory Science, School of Public Health, Biomedical Sciences and Technology, Masinde Muliro University of Science and Technology, Kakamega, Kenya; 4 Department of Medical Biochemistry, School of Medicine, Maseno University, Maseno, Kenya; 5 Department of Internal Medicine, Centre for Global Health, University of New Mexico, Health Sciences Centre, Albuquerque, New Mexico USA; 6 Centre for Global Health Research, Kenya Medical Research Institute, Kisumu, Kenya; 7 African Institute for Development Policy, Nairobi, Kenya

**Keywords:** FcγRs, Susceptibility, Polymorphisms, Malaria anemia

## Abstract

**Background:**

Naturally-acquired immunity to *Plasmodium falciparum* malaria develops after several episodes of infection. Fc gamma receptors (FcγRs) bind to immunoglobulin G (IgG) antibodies and mediate phagocytosis of opsonized microbes, thereby, linking humoral and cellular immunity. FcγR polymorphisms influence binding affinity to IgGs and consequently, can influence clinical malaria outcomes. Specifically, variations in FcγRIIA -131Arg/His, FcγRIIIA-176F/V and FcγRIIIB-NA1/NA2 modulate immune responses through altered binding preferences to IgGs and immune complexes. Differential binding, in turn, changes ability of immune cells to respond to infection through production of inflammatory mediators during *P. falciparum* infection.

**Methods:**

We determined the association between haplotypes of FcγRIIA-131Arg/His, FcγRIIIA-176F/V and FcγRIIIB-NA1/NA2 variants and severe malarial anemia (SMA; hemoglobin < 6.0 g/dL, any density parasitemia) in children (*n* = 274; aged 6–36 months) presenting for their first hospital visit with *P. falciparum* malaria in a holoendemic transmission region of western Kenya. FcγRIIA-131Arg/His and FcγRIIIA-176F/V genotypes were determined using TaqMan® SNP genotyping, while FcγRIIIBNA1/NA2 genotypes were determined using restriction fragment length polymorphism. Hematological and parasitological indices were measured in all study participants.

**Results:**

Carriage of FcγRIIA-131Arg/FcγRIIIA-176F/FcγRIIIBNA2 haplotype was associated with susceptibility to SMA (OR = 1.70; 95% CI; 1.02–2.93; *P =* 0.036), while the FcγRIIA-131His/ FcγRIIIA-176F/ FcγRIIIB NA1 haplotype was marginally associated with enhanced susceptibility to SMA (OR: 1.80, 95% CI; 0.98–3.30, *P =* 0.057) and higher levels of parasitemia (*P =* 0.009). Individual genotypes of FcγRIIA-131Arg/His, FcγRIIIA-176F/V and FcγRIIIB-NA1/NA2 were not associated with susceptibility to SMA.

**Conclusion:**

The study revealed that haplotypes of FcγRs are important in conditioning susceptibility to SMA in immune-naive children from *P. falciparum* holoendemic region of western Kenya.

**Electronic supplementary material:**

The online version of this article (doi:10.1186/s12879-017-2390-0) contains supplementary material, which is available to authorized users.

## Background

In *Plasmodium falciparum* malaria holoendemic transmission regions, such as western Kenya, malaria manifests with a milieu of life-threatening conditions including severe malarial anemia (SMA), metabolic acidosis, high-density parasitemia (≥10,000 parasites/μL), respiratory distress, hypoglycaemia and other infrequent complications such as hypotension [1]. Even though not fully understood, severe clinical malaria is a multi-factorial process involving sequestration of infected red blood cells (iRBCs) in particular organs such as spleen [2], bone marrow suppression leading to dyserythropoiesis [3], and limited, malaria-specific antibody immunity and dysregulation in inflammatory responses [4]. Due to the gradual development of immunity against *P. falciparum* malaria in holoendemic areas, infants and young children suffer the greatest disease burden. The most common clinical manifestation of severe *P. falciparum* malaria infection in pediatric populations of western Kenya is SMA (hemoglobin, Hb < 6.0 g/dL, any density parasitemia) [5].

The binding of immunoglobulin domains to Fc receptors on target cells is important to initiate immunological defense against pathogens including antigen presentation, phagocytosis, cytotoxicity, induction of inflammatory processes and modulation of immune responses [6]. Therefore, Fc gamma receptors (FcγRs) are important in providing a significant link between the humoral and cellular immunity by bridging the interaction between specific antibodies and effector cells [7]. Previous studies demonstrate that polymorphic variability in these receptors is an important determinant of susceptibility to infections [8, 9].

Previous investigations have also shown that the efficacy of the cellular immune response is influenced by FcγR polymorphisms, and consequently, influence clinical outcomes for infectious diseases’ such as malaria [9, 10]. The human FcγRIIA mediates phagocytic function of monocytes, macrophages and neutrophils. The presence of FcγRIIA-131Arg/131His polymorphism affects the binding to the IgG_1_ and IgG_3_ [11]. As reviewed Grant and colleagues [12], FcγRIIA-131His/His homozygotes is associated with higher IgG_2_ levels and protection against high parasitemia and has been considered as protective against blood stage *P. falciparum* infection both in African and Asian populations [13].

FcγRIIIA is an activating receptor with two co-dominantly expressed alleles, the 176 V and the 176F that differ in an amino acid at position 176 in the extracellular domain (valine or phenylalanine, respectively) [14]. Dimorphisms in the amino acid at position 176F/V influences the binding of the immunoglobin G (IgG) subtype, with the 176 V variant having higher binding affinity for monomeric forms of IgG_1_ and IgG_3,_ as compared to the 176F [15] which is potentially important in infectious disease immunity.

On the surface of polymorphonuclear leucocytes, the most abundantly expressed FcγRs is the FcγRIIIB. These receptors exhibits two allotypic forms i.e. neutrophil antigens (NAs) 1 and 2 which differ in minor amino acids at position 65 and 82 in two extra-glycosylation site in NA2 [16, 17] with different binding affinities. The NA2/NA2 allotype is associated with low immunoglobulin-mediated phagocytosis [18, 19]. The phagocytosis of IgG_1_-and IgG_3_-opsonized immune complexes is more efficient on neutrophils bearing FcγRIIIB-NA1 relative to FcγRIIIB-NA2 [18].

A number of genetic association studies have provided evidence that polymorphic variation in FcγRs have a strong effect on susceptibility to inflammatory mediated diseases [20–24]. Even though FcγRs are important in the immune response to infection, the effect of its haplotypes on susceptibility to SMA in immune-naїve children remain largely undetermined. In the present study, we determined the association between FcγRIIA, IIIA and IIIB haplotypes and SMA, and the influence of these haplotypes on peripheral parasite burden during acute falciparum infections in an extensively phenotyped cohort of children from a *P. falciparum* holoendemic transmission area western in Kenya.

## Methods

### Study site

The study was conducted at Siaya County Referral Hospital (SCRH), western Kenya, a *P. falciparum* holoendemic transmission region [25]. Over 98% of the inhabitants are from the Luo ethnic tribe, hence providing a homogenous population for immuno-genetic studies. Falciparum malaria prevalence is ~83% in children aged <4 years, with severe disease manifesting as SMA (Hb < 6.0 g/dL) with or without high-density parasitemia (HDP; ≥10,000 parasites/μL of blood) [5].

### Study participants

Children [*n* = 274, aged 6–36 months] of both sexes were recruited at SCRH during their initial hospitalization for treatment of malaria. Recruitment followed a two-phase tier of screening and enrolment. The parent/legal guardian of the child received detailed explanation of the study. Enrollment decisions were made after initial HIV-1 screening of the child and a signed informed consent, which included authority to publish the findings. Questionnaires and written informed consent were administered in the language of choice (i.e. English, Kiswahili or Dholuo). Children with acute malaria were stratified into two categories: non-severe malarial anemia (non-SMA) group defined as a positive smear for asexual *P. falciparum* parasitemia (of any density) and Hb ≥ 6.0 g/dL; and SMA group defined by a positive smear for asexual *P. falciparum* parasitemia (of any density) and Hb < 6.0 g/dL [25]. Venous blood samples (<3.0 mL) were collected into EDTA-containing vacutainer tubes at the time of enrollment, prior to any treatment interventions or supportive care. Blood samples were used for malaria diagnosis, complete hematological profile measurements, HIV testing, bacterial culture and genetic analyses. Children were excluded from the study for any one of the following reasons; children with CM (a rare occurrence in this holoendemic area); clinical evidence of acute respiratory infection; and prior hospitalization. Participants were treated according to the Ministry of Health (MOH)-Kenya guidelines. This included the administration of oral artemether/lumefantrine (Coartem®) for uncomplicated malaria and intravenous quinine (and when indicted, blood transfusion) for severe malaria.

### Laboratory procedures

Hemoglobin levels and complete blood counts were determined using the Beckman Coulter ACT diff2™ (Beckman-Counter Corporation, Miami, FL, USA). To determine parasitemia, 10% Giemsa-stained thick and thin blood smears were prepared and examined under a microscope on high power magnification. *P. falciparum* parasites per 300 white blood cells (WBCs) were determined, and parasitemia (per μL) was estimated using the total WBC count. In order to delineate severe anemia caused by malaria versus other anemia-promoting conditions, human immunodeficiency virus (HIV)-1, bacteremia and sickle-cell trait (HbAS) were determined in all study participants. The effect of these parameters on disease severity was controlled for during in all regression models. Pre- and post-test HIV counseling was provided for all participants. HIV-1 exposure and infection were determined serologically (i.e., Unigold™ and Determine™) and discordant results confirmed through HIV-1 proviral DNA PCR testing, according to previously published methods [26]. Bacteremia was determined using the Wampole Isostat Pediatric 1.5 system (Wamploe Laboratories, Town, Country). The presence of the sickle cell trait (HbAS) was determined by cellulose acetate electrophoresis (Helena Bio-Sciences, Oxford, United Kingdom) while G6PD deficiency was determined by fluorescent spot test using the manufacturer’s methods (Trinity Biotech Plc., Bray, Ireland).

### Genotyping of FcγRs polymorphisms

Blood spots were made on FTA Classic® cards (Whatman Inc., Clifton, NJ, USA), air-dried, and stored at room temperature until used for DNA extraction. DNA was extracted using the Gentra System (Gentra System Inc., Minneapolis, MN, USA) based on the manufacturer’s instructions. The FcγRIIA-131Arg/His (rs1801274, assay ID: C__9077561_20) and FcγRIIIA -176F/V (rs396991, assay ID: C__25815666_10) polymorphisms were genotyped using the high-throughput TaqMan® 5′ allelic discrimination Assay-By-Design method, according to the manufacturer’s instructions (Applied Biosystems, Foster City, CA, USA), while the FcγRIIIB-NA1/NA2 genotyping for the rs448740 (N65S) and rs147574249 (N82D) was performed according to a previously described RFLP method [27].

### Data analyses

SPSS® statistical software package version 20.0 (IBM SPSS Inc., Chicago, IL, USA) was used for all statistical analyses. Chi-square analysis was used to examine differences between proportions. Mann-Whitney U test was used for comparisons of demographic and clinical characteristics between the two clinical groups. The association between FcγRIIA-131Arg/His, FcγRIIIA-176F/V and FcγRIIIB-NA1/NA2 genotypes, haplotypes and SMA was determined using bivariate logistic regression analysis controlling for confounding effects of age, gender, co-infections (bacteremia and HIV-1), G6PD deficiency, and sickle cell trait (HbAS). Student’s *t*-test was used to determine differences in the levels of parasitemia between the carriage and non-carriage of the haplotypes. Levels of parasitemia were log-transformed to normal distribution. FcγRIIA-131Arg/His, FcγRIIIA-176F/V and FcγRIIIB-NA1/NA2 allele frequencies, consistency and/or deviations from Hardy-Weinberg Equilibrium (HWE) were determined using web-based site emerald.tufts.eduAQ3/~court01/Documents/Court%20lab%20-%20HW. Statistical significance was set at *P* ≤ 0.05.

## Results

### Demographic clinical and laboratory characteristics of study participants

We conducted a cross-sectional analysis of children (*n* = 274, aged 6–36 months) presenting with acute *P. falciparum* malaria (any density parasitemia) (See Additional file 1). Clinically, the study participants were classified into two categories based on a previous study in an age- and geographically-defined reference population from western Kenya [25], i.e., severe malaria anemia (SMA; Hb < 6.0 g/dL; *n* = 114) and non-SMA (Hb ≥ 6.0 g/dL, *n* = 160). There were more males in the non-SMA category compared to the SMA group (*P =* 0.039). Children with SMA were younger (age in months) [median (IQR); 8.0 (7.00)] than those in the non-SMA group [median (IQR); 13.5 (8.80)], *P <* 0.001. Parasitemia values (log_10_ of parasites/μL) was comparable between the study groups, SMA [mean (SEM); 4.09 (±0.07)] and non-SMA [mean (SEM); 4.24 (±0.06)], *P =* 0.088). The proportion of participants with high-density parasitemia (HDP) was also comparable between the clinical groups (62.3% in SMA and 71.9% in non-SMA, *P =* 0.094). Similarly, there was no difference in body temperature (°C) between the study groups, SMA [median (IQR); 38.0 (1.20)] and non-SMA [median, (IQR), [38.0; (1.40)], respectively, *P =* 0.430. Further analysis revealed that children with SMA had higher respiration rate (breaths/min), [median, (IQR); 32.0, (12.00)] than non-SMA, [median, (IQR); 26.0, (14.00)], *P* < 0.001. Analysis of hematological parameters revealed that red blood cells counts (RCBs × 10^12^/μL) were higher in children with non-SMA [median, (IQR); 3.72, (1.16)] than those with SMA, [median, (IQR); 2.20, (0.86)], *P* < 0.001. The SMA group were also characterized by elevated levels of white blood cells counts (WBC × 10^3^/uL) [median (IQR); 13.50 (8.80)] relative to the non-SMA group [median (IQR); 10.95 (5.90)], *P* < 0.001. The platelet counts (platelets ×10^3^/μL) were lower in children with SMA, [median, (IQR), 150.00 (93.00)] as compared to the non-SMA, [median, (IQR), 170.00 (13.10)], *P =* 0.025. The distribution of G6PD in SMA and non-SMA were comparable (7.00% in SMA and 7.50% in non-SMA, *P* = 0.880). Similarly, the distribution of those with sickle cell trait in SMA and non-SMA were comparable (SMA 19.30% while non-SMA 28.70% respectively, *P* = 0.074). These results are presented on Table 1.

**Table 1:** Demographic clinical and laboratory characteristics of study participants

Characteristics	Clinical groups		
	SMA	non-SMA	*P-*value
	(Hb < 6.0 g/dL)	(Hb ≥ 6.0 g/dL)	
	*n* = 114	*n* = 160	
Sex, n (%)			
Male	49 (43.00)	89 (55.40)	**0.039** ^a^
Female	65 (57.00)	71 (44.60)	
Age, (months)	8.0 (7.00)	13.5 (8.80)	**<0.001** ^b^
Log_10_ of parasitemia	4.09 (±0.07)	4.24 (±0.06)	0.088^c^
HDP (≥10, 000 parasites/μL)	71/114 (62.3)	115/160 (71.9)	0.094^a^
Temperature, (°C)	38.0 (1.20)	38.0 (1.40)	0.430^b^
Respiration rate, (breaths/min)	32.0 (12.00)	26.0 (14.00)	**<0.001** ^b^
Haematological indices			
Hemoglobin, g/dL	5.00 (1.00)	7.95 (3.00)	**<0.001** ^b^
Hematocrit, %	15.90 (4.30)	25.00 (7.40)	**<0.001** ^b^
RBC, (× 10^12^/μL)	2.20 (0.86)	3.72 (1.16)	**<0.001** ^b^
RDW, (%)	23.00 (5.20)	20.45 (4.40)	**<0.001** ^b^
WBC (×10^3^/uL)	13.50 (8.80)	10.95 (5.90)	**<0.001** ^b^
Platelet Counts (×10^3^/μL)	150.00 (93.00)	170.00 (13.10)	**0.025** ^b^
Genetic characteristics			
G6PD n (%)	8 (7.00)	12 (7.50)	0.880
Sickle cell trait, n (%)	22 (19.30)	46 (28.70)	0.074

Data are presented as the median (interquartile range) and n (%) of children unless stated otherwise. Parasitemic children (*n* = 274 were categorized as SMA (*n* = 114) and non-SMA (*n* = 160) according to modified definition of SMA (Hb < 6.0 g/dL, with any density parasitemia). ^a^Statistical significance was determined by the Chi-square (χ^2^) analysis. ^b^Statistical significance was determined using Mann-Whitney U test. ^c^Statistical significance was determined using Student’s t-test. *Abbreviations*: *G6PD* Glucose-6-Phaspahte dehydrogenase, *HDP* high density parasitemia, RBC-Red blood cells, RDW - Red cell distribution width; WBC-White blood cells. Probability values were considered statistically significant at *P* ≤ 0.05

Values in bold are significant *p*-values at a cut-off of *p*≤0.05

### Distribution of FcγRIIA-131Arg/His, FcγRIIIA-176F/V and FcγRIIIB-NA1/NA2 genotypes in the clinical groups

Chi square (χ^2^) analysis showed that the distributions of the FcγRIIA-131Arg/His, FcγRIIIA-176F/V and FcγRIIIB-NA1/NA2 genotypes were not significantly different between the clinical groups (*P =* 0.226, *P* = 0.162 and *P =* 0.632, respectively) (Table 2). FcγRIIA-131Arg/His genotypes within the SMA group were 30 (26.3%) Arg/Arg, 59 (51.8%) Arg/His and 25 (21.9%) His/His. Consistency with Hardy-Weinberg Equilibrium (HWE) in the SMA group for FcγRIIA-131Arg/His was observed (χ^2^ = 0.15, *P =* 0.692). FcγRIIA-131Arg/His genotypes distribution in non-SMA were 39 (24.3%) Arg/Arg, 71 (44.4.0%) Arg/Hist and 50 (31.1%) His/His. Frequencies of the genotypes in non-SMA showed deviation from HWE (χ^2^ = 4.92, *P =* 0.027). The overall genotype distribution for the FcγRIIA-131Arg/His did not deviate from HWE (χ^2^ = 0.703, *P =* 0.402) with an overall variant allele frequency of the FcγRIIA-131Arg/His at 0.49 (Arg). The genotypic distribution of the FcγRIIIA-176 F/V in SMA group was 61 (53.5%) FF, 45 (39.5%) FV and 8 (7.0%) VV. The distribution of these genotypes in SMA showed consistency with HWE (χ^2^ = 0.006, *P =* 0.939). Within the non-SMA group, the distributions was 77 (48.1%) FF, 60 (37.5%) FV and 23 (14.4%) for VV and the genotypes showed consistency with HWE (χ^2^ = 3.774, *P =* 0.052). The distribution of these genotypes in overall population showed consistency with HWE (χ^2^ = 2.510, *P =* 0.113) and had an overall mutant allele frequency of 0.30 (V). FcγRIIIB-NA1/NA2 genotypes distribution in the SMA group were 6 (5.3%) NA1, 73 (64.0%) NA1/NA2 and 35 (30.7%) NA2, while in non-SMA there was 8 (5.0%) NA1, 94 (58.8%) NA1/NA2 and 58 (36.2%) NA2. The distributions of the genotypes in both SMA and non-SMA revealed deviation from HWE normality (χ^2^ = 15.549, *P* < 0.001, and χ^2^ = 14.608, *P* < 0.001, respectively). In addition, HWE deviation was revealed by the FcγRIIIB-NA1/NA2 genotypes’ distribution considering the whole study group (χ^2^ = 29.47, *P* < 0.001) with variant allele frequency of 0.36 (NA1), Table 2.

**Table 2:** Distribution of FcγRIIA-131Arg/His, FcγRIIIA-176F/V and FcγRIIIB-NA1/NA2 genotypes within the study groups

	N (%) with genotype in group^a^			HWE, *P*-value (SMA + non-SMA*)* ^c^
Genotypes	SMA (Hb < 6.0 g/dL) (*n* = 114)	Non-SMA (Hb ≥ 6.0 g/dL) (*n* = 160)	*P*-value ^b^	
FcγRIIA-131Arg/His				
Arg/Arg, n (%)	30 (26.3)	39 (24.3)		
Arg/His n (%)	59 (51.8)	71 (44.4)	0.226^b^	0.402^b^
His/His, n (%)	25 (21.9)	50 (31.3)		
X(His) = 0.48				
FcγRIIIA-176 F/V				
FF, n (%)	61 (53.5)	77 (48.1)		
FV, n (%)	45 (39.5)	60 (37.5)	0.162^b^	0.113^b^
VV, n (%)	8 (7.0)	23 (14.4)		
X(V) = 0.30				
FcγRIIIB-NA1/NA2				
NA1/NA1	6 (5.3)	8 (5.0)		
NA1/NA2	73 (64.0)	94 (58.8)	0.632^b^	**<0.001** ^b^
NA2/NA2	35 (30.7)	58 (36.2)		
X(NA1) = 0.36				

^a^Data are presented as n (%) of children. Children with parasitemia were categorized on the basis of presence or absence of severe malarial anemia SMA based (defined as Hb < 6.0 g/dL, with any density parasitemia). ^b^Statistical significance determined by χ^2^ analysis. X; the overall minor allele frequency in the study population. ^c^ *HWE* Hardy-Weinberg Equilibrium

Values in bold are significant *p*-values at a cut-off of *p*≤0.05

### Association between FcγRIIA-131Arg/His, FcγRIIIA-176F/V and FcγRIIIB-NA1/NA2 and severe malarial anemia (SMA, Hb < 6.0 g/dL)

We conducted genetic association analysis based on dominant, additive and recessive models of the FcγR polymorphisms. The FcγRIIA-131His/His dominant model did not reveal association with SMA susceptibility (OR = 0.59, 95% CI, 0.33–1.05, *P =* 0.077). Further analysis did not reveal association between SMA using the additive (OR = 1.52, 95% CI, 0.72–2.93, *P* = 0.298) or the recessive model (OR = 0.98, 95% CI, 0.56–1.75, *P* = 0.963). The dominant (OR = 1.27, 95% CI, 0.79–2.10, *P* = 0.343) and the additive (OR = 0.77, 95% CI, 0.63–1.83, *P* = 0.796) model of the FcγRIIIA-176 F/V dimorphism did not show associations with SMA. However, the recessive model of FcγRIIIA-176 F/V showed a trend towards protection against SMA, albeit with marginal significance (OR, 0.43, 95% CI, 0.18–1.02, *P* = 0.056). Analysis of all the genetic models of FcγRIIIB-NA1/NA2 variation did not reveal any association with SMA; dominant [OR = 0.76, 95% CI, 0.44–1.28, *P* = 0.786)], additive [OR = 1.34, 95% CI, 0.78–2.30, *P* = 0.288] and recessive [OR = 1.20, 95% CI 0.36–3.94, *P* = 0.767), Table 3.

**Table 3:** Association between FcγRIIA-131Arg/His, FcγRIIIA-176F/V, FcγRIIIB-NA1/NA2 and severe malarial anemia (SMA, Hb < 6.0 g/dL)

FcγR genotype models			SMA (Hb < 6.0 g/dL)		
	SMA	Non-SMA	OR	95% CI	*P*-value
FcγRIIA-131Arg/His					
Dominant, (His/His, *n* = 75)	25	50	0.59	0.33–1.05	0.077
Additive, (Arg/His, *n* = 130)	59	71	1.52	0.72–2.93	0.298
Recessive, (Arg/Arg, *n* = 69)	30	39	0.98	0.56–1.75	0.963
FcγRIIIA-176 F/V					
Dominant, (F/F, *n* = 138)	61	77	1.27	0.79–2.10	0.343
Additive, (F/V, *n* = 105)	45	60	0.77	0.63–1.83	0.796
Recessive, (*V*/V, *n* = 31)	8	23	0.43	0.18–1.02	**0.056**
FcγRIIIB-NA1/NA2					
Dominant, (NA2/NA2, *n* = 93)	35	58	0.76	0.44–1.28	0.786
Additive, (NA1/NA2, *n* = 167)	73	94	1.34	0.78–2.30	0.288
Recessive, (NA1/NA1, *n* = 14)	6	8	1.20	0.36–3.94	0.767

Children with acute malaria (*n* = 274) were grouped based on SMA (defined as Hb < 6.0 g/dL, with any density parasitemia) [25]. Odds ratios (OR) and 95% confidence intervals (CI) were determined using bivariate logistic regression controlling for age, gender, co-infections (HIV-1 and bacteremia) sickle cell trait (HbAS) and G6PD deficiency. The reference groups in the logistic regression analysis were the absence of the respective models for each genotype. *n* = the number of participants with the respective genotype. *P*-values were considered significant at *P* ≤ 0.05

Values in bold are significant *p*-values at a cut-off of *p*≤0.05

### FcγRIIA-131/FcγRIIIA-176/FcγRIIIB haplotypes distribution within the study groups and association with severe malarial anemia

Prior to performing regression analysis to determine the association between the FcγRIIA-131His/Arg, FcγRIIIA-176F/V and FcγRIIIB-NA1/NA2 haplotypes and SMA, we compared the distribution of the carriage of the haplotypes within the study groups. In total, eight haplotypes were generated after haplotype construction. We selected four common haplotypes with an overall frequency > 8.0% in the whole population. The haplotypes were distributed as follows; FcγRIIA-131Arg/FcγRIIIA-176F/FcγRIIIBNA2, (0.33), FcγRIIA-131His/FcγRIIIA-176F/FcγRIIIBNA1 (0.12), FcγRIIA-131His/FcγRIIIA-176F/FcγRIIIBNA2 (0.17) and FcγRIIA-131His/FcγRIIIA176V/FcγRIIIBNA1 (0.15). Among these four common haplotypes, FcγRIIA-131Arg/FcγRIIIA-176F/FcγRIIIBNA2 haplotype was higher in children with SMA relative to non-SMA group (*P =* 0.044, Table 4). The distributions of the other three haplotypes were comparable between the SMA and non-SMA groups; FcγRIIA-131His/FcγRIIIA-176F/FcγRIIIBNA1 (*P =* 0.104), FcγRIIA-131His/FcγRIIIA-176F/FcγRIIIBNA2 (*P =* 0.269) and FcγRIIA-131His/FcγRIIIA-176 V/FcγRIIIBNA1 (*P =* 0.188, Table 4). Using bivariate logistic regression analysis controlling for age, sex, co-infection (HIV-1 status and bacteremia), sickle cell trait (HbAS) and G6PD deficiency [26, 28–30], we determined the association between carriages of the FcγRIIA-131/FcγRIIIA-176/FcγRIIIB haplotypic structures and severe malaria anemia (SMA; Hb < 6.0 g/dL and any density parasitemia). This analysis revealed that the carriage of the FcγRIIA-131Arg/FcγRIIIA-176F/FcγRIIIBNA2 haplotype was associated with increased risk of severe malaria anemia relative to none carriage (OR = 1.70, 95% CI, 1.02–2.93, *P =* 0.036). Further regression analysis did not show any association between carriage of FcγRIIA-131His/FcγRIIIA-176F/FcγRIIIBNA1 (OR = 1.80, 95% CI, 0.98–3.30, *P =* 0.057), FcγRIIA-131His/FcγRIIIA-176F/FcγRIIIBNA2 (OR = 0.76, 95% CI, 0.44–1.32, *P =* 0.334) and FcγRIIA-131His/FcγRIIIA-176 V/FcγRIIIBNA1 (OR = 0.71, 95% CI, 0.41–1.25, *P =* 0.234) haplotypes and SMA.

**Table 4:** FcγRIIA-131/FcγRIIIA-176/FcγRIIIB haplotypes distribution within the study groups and association with severe malarial anemia

FcγRIIA-131His/Arg, FcγRIIIA-176F/V and FcγRIIIB-NA1/NA2 haplotypes	Study groups		*P*-value*	SMA (Hb < 6.0 g/dL		
	SMA n (%)	non-SMA n (%)		OR	95% CI	*P*-value**
131Arg/176F/NA2 (*n* = 171)	79 (69.3)	92 (57.5)	**0.044**	1.70	1.02–2.93	**0.036**
131His/176F/NA1 (*n* = 59)	30 (26.3)	29 (18.1)	0.104	1.80	0.98–3.30	0.057
131His/176F/NA2 (*n* = 87)	32 (28.1)	55 (34.4)	0.269	0.76	0.44–1.32	0.334
131His/176 V/NA1 (*n* = 79)	28 (24.6)	51 (31.9)	0.188	0.71	0.41–1.25	0.234

Children with acute malaria (*n* = 274) were grouped based on SMA (defined as Hb < 6.0 g/dL, with any density parasitemia) [25]. Odds ratios (OR) and 95% confidence intervals (CI) were determined using bivariate logistic regression controlling for age, gender, co-infections (HIV-1 and bacteremia) sickle cell trait (HbAS), alpha-thalassemia and G6PD deficiency. The reference groups in the regression analysis were the non-carriage of respective haplotypic structures. n; the number of participants with the respective haplotype. n (%); number (percentage) of participants with respective haplotype in each study group. *P-value determined using Chi-square (χ^2^). **P-values determined using logistics regression analysis. All *P*-values were considered statistically significant at *P* ≤ 0.05

Values in bold are significant *p*-values at a cut-off of *p*≤0.05

### Association between FcγRIIA-131Arg/His, FcγRIIIA-176F/V and FcγRIIIBNA1/NA2 haplotypes and parasitemia levels

**Fig. 1:**
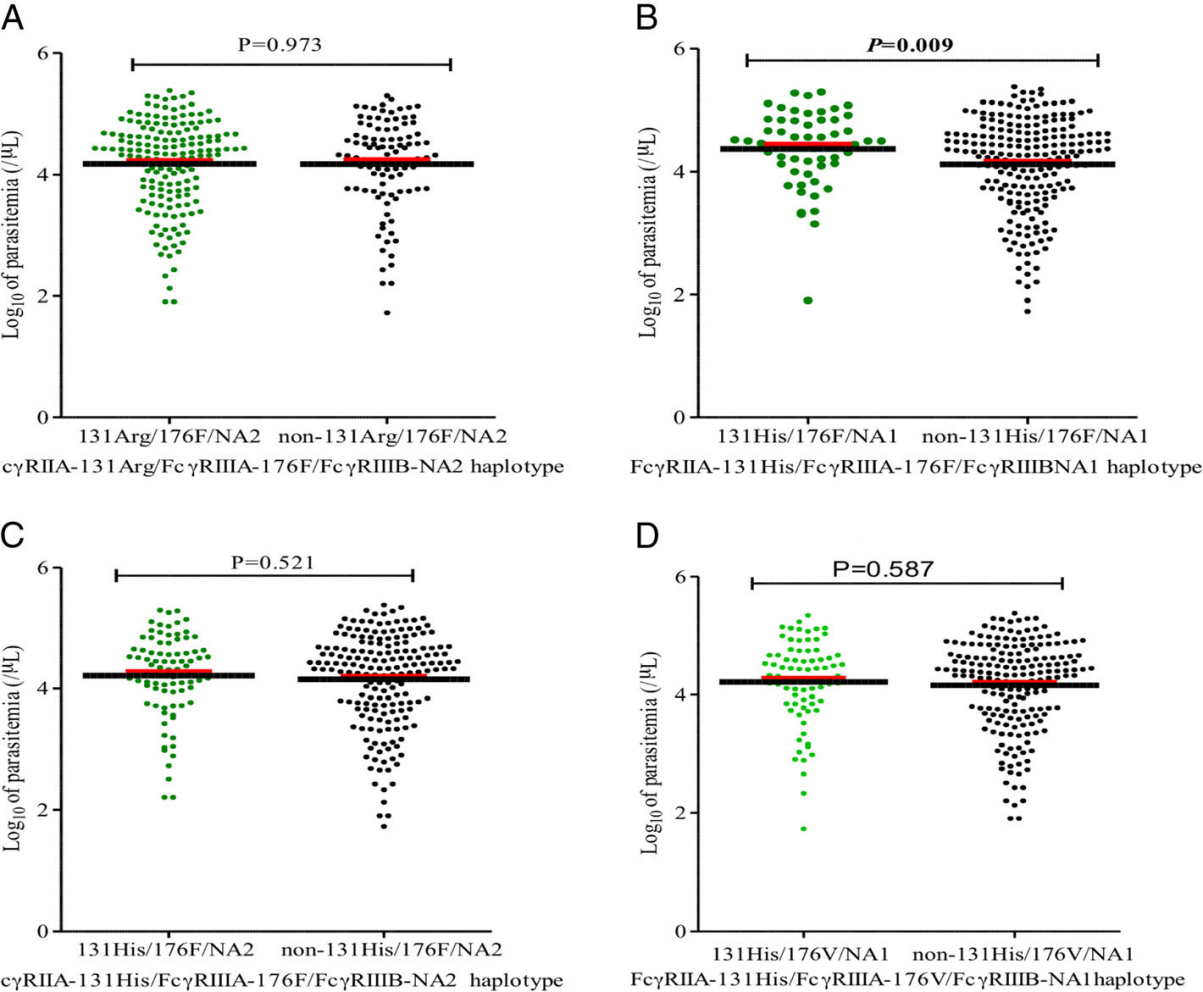
Data are presented as scatter plots for carriage and non-carriage of respective haplotype constructs. The thick *black lines* through the scatters represent mean, while the *red lines* above the mean line represent the standard error of the mean (SEM). The carriage of FcγRIIA-131His/FcγRIIIA-176F/FcγRIIIBNA1 haplotype which was marginally associated with susceptibility to SMA had higher levels of parasitemia (*P =* 0.009). Differences in parasitemia levels were determined using unpaired Student’s t-test with Welsch correction at 95% confidence interval

Since the FcγRs are important determinants in phagocytosis of parasites, we determined if carriage of FcγRs haplotypes was associated with parasitemia levels. Results revealed that carriage of FcγRIIA-131His/FcγRIIIA-176F/FcγRIIIBNA1 haplotype [mean (SEM); 4.37 (± 0.079), *n* = 59] relative to non-carriage [mean (SEM); 4.12 (± 0.052), *n* = 215], *P =* 0.009), was associated with higher parasitemia. Additional analysis showed that the level of parasitemia was comparable between the carriage and non-carriage of FcγRIIA-131Arg/FcγRIIIA-176F/FcγRIIIBNA2 haplotype [mean (SEM); 4.18 (± 0.057), *n* = 171] versus non-carriage [mean (SEM); 4.17 (± 0.074), *n* = 103], *P =* 0.973) and FcγRIIA-131His/FcγRIIIA-176F/FcγRIIIBNA2 [mean (SEM); 4.23 (± 0.073), *n* = 87] versus non-carriage [mean (SEM); 4.16 (± 0.056), *n* = 187, *P =* 0.521]. Further analysis also showed that the level of parasitemia was also comparable between those with FcγRIIA-131His/FcγRIIIA-176 V/FcγRIIIBNA1 haplotype [mean (SEM); 4.21 (± 0.079), *n* = 79] versus those without the haplotype [mean (SEM); 4.16 (± 0.096), *n* = 195], *P =* 0.587), Fig. 1(a-d).

## Discussion

Based on the observations that Fc gamma receptors (FcγRs) are important contributory factors for infectious disease immuno-pathogenesis, the association between the FcγRIIA-131Arg/His, FcγRIIIA-176F/V and FcγRIIIB-NA1/NA2 polymorphisms and pediatric severe malaria anemia (SMA; Hb < 6.0 g/dL, any density parasitemia) was determined. We further assessed whether the carriage of different haplotypes of FcγRs were associated with parasite levels in *P. falciparum* infections. The current study demonstrated that the FcγRIIA-131Arg/ FcγRIIIA-176F/FcγRIIIBNA2 haplotype was associated with an increased susceptibility to SMA, while the FcγRIIA-131Arg/ FcγRIIIA-176F/ FcγRIIIBNA1 haplotype was associated with increased levels of circulating parasites during infection. However, there was no association between the individual genotypes and SMA in this pediatric population from western Kenya.

The FcγRs constitute a crucial arm of host immune defense against extracellular challenges by infectious agents through engagement of IgGs to enable innate immune effectors cells carry out phagocytosis and other downstream processes leading to immunity [14, 31]. Some polymorphisms in the FcγRs have been identified as genetic determinants of susceptibility to infectious diseases [21, 32]. The FcγRIIA-131Arg/His polymorphism leads to Histidine to Arginine change at 131 located at its second extracellular immunoglobulin-like domain [8, 33]. The FcγRIIA-31His/His has efficient binding to IgG_2_ as opposed to FcγRIIA-131Arg/Arg. In addition, the IgG_2_ and IgG_3_ antibodies have been shown to confer resistance to malaria by some studies [34, 35]. In our current study, however, we did not find any association between FcγRIIA-131Arg/His polymorphism and SMA. An earlier study [23] in Ghanaian children demonstrated that FcγRIIA-131His/His was associated with an increased risk of severe malaria anemia, but not cerebral malaria or any other malarial complication. Of note is the fact that a number of studies have shown contradictory results on the actual role of this variant on malarial disease severity [36, 37]. These discrepancies may be attributed to clinical definitions of malaria, different genetic backgrounds from ethnic diversity and overall sample (population) size in previous studies.

The FcγRIIIA-176F/V gene displays a functional allelic polymorphism that generates allotypes exhibiting different receptor properties [38]. Our study revealed no association between the FcγRIIIA-176F/V polymorphism and susceptibility to SMA in this pediatric population. This may imply that this particular variant is not independently associated with susceptibility to SMA which is consistent with our previous study involving the combined effect of toll-like receptor 9 and FcγRIIIA polymorphisms [39]. The FcγRIIIB is a C-terminus linked glycosylphosphatidylinositol (GPI) moiety anchored receptor, exclusively expressed on neutrophils with three characterized allotypes i.e. human neutrophil antigen (HNA-1a or NA1, HNA-1b or NA2 and HNA-1c or SH) [27]. The NA variants, NA1 and NA2, are a product of five non-synonymous SNPs in the first Ig-like domain, with an asparagine to serine switch at amino acid position 65 resulting in glycosylation and reduced affinity in the NA2 allele [19, 38]. In the current study, we did not observe an association between either the NA1 or NA2 allotypes and susceptibility to SMA using common genetic models i.e., dominant, additive and recessive models. However, in Ghanaian children aged 1 to 12 years, the FcγRIIIB-NA2 was associated with susceptibility to clinical malaria [40]. In a different study of malaria patients in Thailand, the FcγRIIIB-NA2 allotype was associated with cerebral malaria, but not other forms of severe malaria [21]. Given the differences in findings from different populations and a diversity of clinical manifestations associated with malaria, the exact role of FcγRIIIB-NA2 in mediating outcome of malarial disease remains to be further explored.

It is important to note that FcγRs function synergistically, especially via crosslinking, resulting in phagocytosis of immunoglobulin-opsonized immune complexes or through stimulation of neutrophil granulation leading to production of reactive oxygen species (ROS) [41, 42]. Moreover, the additive and interaction effects of host genotype and infection affect malaria outcome [43] in malaria. In the current study, haplotypic analysis revealed that carriage of the FcγRIIA-131Arg/FcγRIIIA-176F/FcγRIIIBNA2 haplotype was associated with susceptibility to SMA. This is not surprising given that the haplotype had a higher frequency in the SMA group relative to the non-SMA group. Consistent with these observations, previous studies have demonstrated that the FcγR-131Arg/Arg is associated with low phagocytic activity and poor immune complex clearance [33], which may imply that its inheritance as a haplotype, together with FcγRIIIA-176F and the FcγRIIIBNA2 allotypes, impart decreased cellular responses to IgG-mediated stimulation [15, 18], and subsequently, susceptibility to SMA. Although the exact mechanisms through which the FcγRIIA-131Arg/FcγRIIIA-176F/FcγRIIIBNA2 haplotype result in severe malaria susceptibility were not evaluated in the current study, it is scientifically plausible to propose that carriage of the haplotype may lead to a reduced crosslinking in neutrophils, hence low phagocytic activity resulting in reduced antibody dependent respiratory burst (ADRB), a mechanism by which neutrophils provide protection against clinical malaria [44–46]. Moreover, the FcγRIIA-131Arg/Arg, FcγRIIIA-176F/F and FcγRIIIB-NA2 allotypes are associated with low binding to cytophilic antibodies, which have been shown to play major roles in ADRB [47, 48]. Taken together, the FcγRIIA-131Arg/FcγRIIIA-176F/FcγRIIIBNA2 haplotype may culminate in a reduced protective inflammatory response leading to enhanced susceptibility in children with SMA.

The finding that the FcγRIIA-131His/FcγRIIIA-176F/FcγRIIIBNA1 haplotype was associated with higher parasitemia levels is fascinating given the fact that the FcγRIIA-131His/His and FcγRIIIB-NA1 allotypes in this haplotype construct are associated with effective binding to cytophillic IgGs [49], leading to clearance of opsonized parasites as opposed to the FcγRIIIA-176 F/F. One possible explanation for this observation could be that high levels of parasitemia in the haplotype may be associated with the diluting effect of the FcγRIIA-176F allele, which has a low binding to cytophylic antibodies [15], and hence reduced clearance of parasites. However, it is worth noting that FcγRIIIA binding of IgG is important in induction of natural killer (NK) cells stimulatory properties which results in release of pro-inflammatory mediators, such as IL-1β, interferon-γ and tumor necrosis factor-α [50] whose imbalances have been implicated in pathogenesis of clinical malaria in children.

Differences in the allelic frequencies of the FcγRs SNPs observed in the current study likely suggest their indirect influence on malaria susceptibility and pathogenesis in the current population. The deviation from HWE of FcγRIIIB NA1/NA2 genotypes in the current study remains consistent with the results of FcγRIIIB genetic polymorphisms performed in our previous reporting in which we included 528 children [22]. It is likely that the observed NA1/NA2 genotype frequencies were in part due to consanguinity, however, this effect was not determined in the current study population. As much as HWE inconsistency may be due to genotyping errors [51], it is worth noting that the likelihood of this error was significantly reduced since in our previous population [22] we genotyped both FcγRIIA -131Arg/His and FcγRIIIB-NA1/NA2 using RFLP method in which the genotype frequencies were comparable to those in the current population in which TaqMan genotyping was used for FcγRIIA -131Arg/His. We thus hypothesize that the observed HWE deviation in FcγRIIIB could be due to unidentified mutation likely resulting from disease-related evolutionary selection pressure by *P. falciparum* (and potentially by other infectious disease in the population) that does not affect the neighboring FcγRIIA and FcγRIIIA genes. This, however, remains to be determined most preferably by whole genome sequencing so as to develop a conclusive explanation.

In summary, the current study demonstrates that FcγRs haplotypes, but not individual genotypes are associated with malarial disease severity, demonstrating the combinatorial effects of FcγRs on influencing clinical malaria outcomes. Future studies aimed at longitudinally measuring immune complexes over time will help to delineate the important role of FcγR haplotypes on susceptibility to severe malaria in pediatric populations.

## Additional file

Additional file 1: These are details of the raw data for the study participants (N=274) used in the analyses of results presented in the current paper. (XLS 222 kb)

## Abbreviations

ADRB: Antibody dependent respiratory burst
CM: Cerebral malaria
FcgR: Fc gamma receptor
G6PD: Glucose-6-phosphate dehydrogenase
Hb: Hemoglobin
HbAS: Hemoglobin AS type
HDP: High density parasitemia
HWE: Hardy Weinberg equilibrium
IgG: Immunoglobulin
IQR: Interquartile range
MOH: Ministry of Health
NA: Neutrophil antigen
NK: Natural killer cells
SEM: Standard error of mean
SMA: Severe malarial anemia
SNP: Single nucleotide polymorphisms
WBCs: White blood cells
